# 

**Published:** 1911-07-15

**Authors:** 


### ANNOUNCEMENTS.

### New York 7th and 8th District Dental Societies.

These Societies will hold a joint meeting at the Hotel Statler, in Buffalo, November 9 to 11, 1911. This is one of the active New York organizations and can be depended on to present a programme worth while, and this occasion will doubtless be no exception.

### The Dental Educational Council of America.

The second annual meeting of the Dental Educational Council of America will be held at the Colonial Hotel, Cleveland, Ohio, Monday, July 24th, at 5:00 P. M. An advisory conference will also be held in the evening of the same day at 7:30 o'clock, to which delegates from the following organizations are cordially invited:

National Dental Association
State Dental Societies.
Local City Dental Societies.
Editors of American Dental Journals.
State Boards of Dental Examiners.
American Dental Colleges.

The purpose of this advisory conference is to listen to the reading of the annual report of the Secretary and to invite a study and discussion of the problems of Dental Education.

It is hoped that the friends of dentistry will attend this meeting, and to this end your co-operation is respectfully requested.

Chas. P. Pruyn, *President.*

Richard Summa, *Vice Persident.*

Henry L. Banzhaf, *Secy.-Treas.*

June 21, 1911.

Office of Secretary.

120 Wisconsin St.,

Milwaukee, Wis.

